# The role of the brain–gut–microbiota axis in psychology: The importance of considering gut microbiota in the development, perpetuation, and treatment of psychological disorders

**DOI:** 10.1002/brb3.1408

**Published:** 2019-09-30

**Authors:** Michael Ganci, Emra Suleyman, Henry Butt, Michelle Ball

**Affiliations:** ^1^ Psychology Department Institute for Health and Sport Victoria University Melbourne Vic. Australia; ^2^ Bioscreen Yarraville (Aust) Pty Ltd Melbourne Vic. Australia; ^3^ Melbourne University Melbourne Vic. Australia

**Keywords:** allostatic load, gut microbiota, precipitating factors, predisposing factors, protective factors, psychology

## Abstract

**Introduction:**

The prevalence of psychological disorders remains stable despite steady increases in pharmacological treatments suggesting the need for auxiliary treatment options. Consideration of the brain–gut–microbiota axis (BGMA) has made inroads into reconceptualizing psychological illness from a more holistic perspective. While our understanding of the precise role of gut microbiota (GM) in psychological illness is in its infancy, it represents an attractive target for novel interventions.

**Method:**

An extensive review of relevant literature was undertaken.

**Results:**

Gut microbiota are proposed to directly and indirectly influence mood, cognition, and behavior which are key components of mental health. This paper outlines how GM may be implicated in psychological disorders from etiology through to treatment and prevention using the Four P model of case formulation.

**Conclusion:**

Moving forward, integration of GM into the conceptualization and treatment of psychological illness will require the discipline of psychology to undergo a significant paradigm shift. While the importance of the GM in psychological well‐being must be respected, it is not proposed to be a panacea, but instead, an additional arm to a multidisciplinary approach to treatment and prevention.

## Graphical abstract

Gut microbiota are proposed to influence mood, cognition, and behavior both directly and indirectly. This offers a new approach to conceptualizing psychological disorders from predisposition through to treatment. While not suggesting that targeting the gut microbiota is the panacea to all psychological conditions, it does offer an exciting new arm to a holistic and multidisciplinary approach to promoting psychological well‐being.

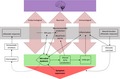

## 1 INTRODUCTION

Burgeoning research regarding the role of the gut and its microbial inhabitants in the pathophysiology of psychological illness is gaining momentum. Early evidence points to gut microbiota (GM) as a possible missing link in the conceptualization and treatment of psychological illness that sees disparities between conventional treatment methods and prevalence rates. When discussing the role of GM in behavior, health, and disease, it is important to pay respect to the intertwined coevolution between humans and our resident microbes. It is suggested that the sharp increase in various disease states over the last 50–100 years (Campell, 2014; Linneberg et al., 2000) can be, at least in part, explained by relatively recent dietary and lifestyle changes in the context of human evolution (Broussard & Devkota, 2016). Currently, humans, particularly those in industrialized countries, are living in an environment to which they have not adaptively evolved (Gluckman, Low, Buklijas, Hanson, & Beedle, 2011). An unintended consequence of industrialization, these changes are putting the GM under evolutionary pressure to shift from a previously mutualistic relationship with their human host to a more antagonistic one (Broussard & Devkota, 2016; Quercia et al., 2014). This is due to human evolution requiring significantly more time (Uyeda, Hansen, Arnold, & Pienaar, 2011) compared to single‐celled organisms such as GM that evolve and adapt to environmental and internal states much more rapidly (within as little as 24 hr; David et al., 2014; Wu et al., 2011). These variations in the evolutionary pressures and capabilities of the two components (host and GM) of a single ecosystem (the holobiont; Theis et al., 2016) result in systemic disharmony.

This systemic disharmony leads to symptomatology and disease states that are the primary target for intervention in current medical models, which precludes effective etiology‐focused prevention (Marvasti & Stafford, 2012). This is exacerbated given that current medical models are heavily skewed toward treatment over prevention (Singh, 2010). While there is disagreement over whether the occurrence of common mental disorders are increasing or whether their prevalence remains consistent (Friedrich, 2017; Harvey et al., 2017), the consumption of antidepressant drugs doubled in most, if not all, Organisation for Economic Co‐operation and Development (OECD) countries between 2000 and 2015 (OECD, 2017). Despite this, depression has recently taken over as the leading cause of disability worldwide with anxiety also in the top 10 leading causes of disability (World Health Organisation (WHO) 2017). Furthermore, subclinical psychological symptoms that do not meet the full Diagnostic and Statistical Manual for Mental Disorders (DSM) or International Classification of Diseases (ICD) diagnostic criteria are also prevalent (Angst, Merikangas, & Preisig, 1997; Haller, Cramer, Lauche, Gass, & Dobos, 2014; Mathieson, Collings, & Dowell, 2009). These subclinical symptoms lead to impairment in psychosocial and work functioning, are a major determinant of sick leave, contribute to the use of psychotropic drugs and primary health care services, and reduce quality of life (Haller et al., 2014; Johansen & Dittrich, 2013; Mendlowicz & Stein, 2000).

The conceptualization of the human body as being made up of separate and distinguishable systems is likely to have contributed to our current poor appreciation of the complexity of mechanisms that underlie the etiology and progression of disease. Psychology, just like many other healthcare professions and medical science disciplines, specialize and focus on specific body systems. For example, treatment of psychological symptoms and disorders tends to be central nervous system (CNS)‐centric with the brain being the principle target for both psychotherapy and psychopharmacology while peripheral systems, such as the gut, receive little attention. While there is no doubt that specialization has its benefits and has contributed to the progression of medical knowledge, it also has its drawbacks. Operating within these constrictive distinctions imposed by disciplinary specialization means that the complex interplay between various body systems essential to proper functioning is overlooked. The true etiology of disease is likely to come from dysregulations in this interplay which may also go some way to explaining high levels of comorbidity, particularly between functional gastrointestinal disorders such as IBS and psychological conditions (Garakani et al., 2003; Lee et al., 2015).

Within the field of psychology, an integral part of intervention is case formulation. Essentially, case formulation involves the synthesis of information about a patient (typically gained through clinical interviews and formalized cognitive testing) in a meaningful way to facilitate the development of a treatment plan. A commonly used model in structuring a case formulation is that of the Four Ps which represent the predisposing, precipitating, perpetuating, and protective factors related to a client's presenting problem and thus the targets of psychological intervention. As information is drawn from clinical interviews and cognitive testing, the functioning of a client's gut is seldom considered in their formulation; thus, important diagnostic and treatment options may be missed. It is the contention of this paper to demonstrate that GM are intimately linked with each of these four pillars of psychological intervention and thus each stage of disease, from etiology through to treatment and prevention. This paper adds to the burgeoning research into the brain–gut–microbiota axis (BGMA; Kelly, Clarke, Cryan, & Dinan, 2016) demonstrating that the discipline of psychology is on the cusp of a significant paradigm shift, moving away from CNS‐centric approaches toward a more holistic conceptualization of health and disease which integrates other body systems. We echo the sentiment of Allen, Dinan, Clarke, and Cryan (2017) who call for a challenge to the reductionist approaches in psychology in favor of a multidisciplinary approach to conceptualizing and treating psychological disorders. In taking the unique approach of the Four P model of case formulation, this paper intends to review existing research on associations between GM and psychological outcomes, compiling it in a way that is more accessible to psychologists, especially those who have little previous knowledge regarding the BGMA.

### 1.1 The brain–gut–microbiota axis

The BGMA is increasingly being recognized as playing an important role in homeostasis and consequentially, health and disease states (e.g., Mu, Yang, & Zhu, 2016; Rea, Dinan, & Cryan, 2016). The BGMA refers to the relationship between the brain and the gut while acknowledging the important moderating role of GM (e.g., Carabotti, Scirocco, Maselli, & Severi, 2015; Grenham, Clarke, Cryan, & Dinan, 2011). Largely recognized as a bidirectional relationship (e.g., Rhee, Pothoulakis, & Mayer, 2009), research continues to uncover the true complexities of this communication network which Rea et al. (2016) more accurately define as a multidirectional relationship. It is multidirectional in the sense that each component of this extensive communication network has the ability to moderate and manipulate the function of the other systems involved. Communication between the brain and the gut is maintained via a complex network including the CNS, autonomic nervous system (ANS), enteric nervous system (ENS), hypothalamic–pituitary–adrenal (HPA) axis, neural, endocrine and immune systems (e.g., Carabotti et al., 2015; Cryan & Dinan, 2012; Mayer, 2011; Moloney, Desbonnet, Clarke, Dinan, & Cryan, 2014). Essentially, the BGMA provides a network for signals from the brain to influence the motor, sensory, and secretory functions of the gut while simultaneously allowing signals and metabolites from the GM to influence brain development, biochemistry, function, and behavior (e.g., Cryan & O'Mahony, 2011; Grenham et al., 2011; Marques et al., 2014). This communication system presents an exciting and novel target for psychological intervention, providing a deeper understanding of the biological underpinnings of psychological illnesses. It is hoped that developing a greater understanding of the BGMA as it relates to the Four Ps of case formulation will provide psychologists with an additional tool in the treatment of their clients.

### 1.2 Is diet the chicken, or the egg?

The relationship between diet and GM is an intriguing one, ironically reminiscent of the idiom regarding which came first, the chicken or the egg. Once thought to be unidirectional (diet influencing the composition of the microbiota), recent evidence suggests the relationship could in fact be bidirectional, with microbes also being able to influence food choice and dietary‐related behaviors (Alcock, Maley, & Aktipis, 2014).

Diet is arguably one of the most important environmental factors in shaping the composition and metabolic activities of GM (De Filippo et al., 2017; Garcia‐Mantrana, Selma‐Royo, Alcantara, & Collado, 2018; Voreades, Kozil, & Weir, 2014). As such, diet must be taken into consideration when discussing potential interventions involving GM. An extensive review of the relationship between diet and GM is beyond the scope of this paper, but can be found elsewhere (e.g., Sheflin, Melby, Carbonero, & Weir, 2017; Singh et al., 2017; Wu et al., 2011). Here, diet is discussed insofar as to highlight to psychologists the importance of this environmental factor in the formulation and treatment of their client's presenting problem. While it is not being suggested that psychologists become well versed in dietetics, gathering general information on a client's diet may provide further insight into the development and perpetuation of their presenting problem, and presents as an additional arm to a multidisciplinary and holistic treatment approach.

Both the content and diversity of an individual's diet are believed to be important in maintaining a well‐balanced GM (Heiman & Greenway, 2016; Oriach, Robertson, Stanton, Cryan, & Dinan, 2016). Diet quality has also been highlighted as a potential risk or protective factor for conditions such as depression (Jacka et al., 2017; Koopman & El Aidy, 2017; Lai et al., 2014). In regards to dietary content, the industrial revolution saw a significant increase in highly processed cereals rich in carbohydrates, refined sugars, sodium, omega‐6, and trans‐fatty acids. Concurrently, potassium, complex carbohydrates, fiber, omega‐3, and unsaturated fatty acids were considerably reduced (Rubio‐Ruiz, Peredo‐Escárcega, Cano‐Martínez, & Guarner‐Lans, 2015). These changes are reflective of what is today termed the “Western‐style diet,” one that has been shown to impair immune function and promote inflammation (Myles, 2014). This is concerning given that inflammation is believed to underlie and perpetuate many, if not all, psychological and neurodegenerative illnesses (Almond, 2013; Miller & Raison, 2016; Rea et al., 2016). Worryingly, dietary diversity has been further reduced over the past 50 years with an ever increasing preference for convenience and taste (Glanz, Basil, Maibach, Goldberg, & Snyder, 1998; Heiman & Greenway, 2016; Poti, Mendez, Ng, & Popkin, 2015). Essentially, this means that current human diets are not providing GM with the resources they require to perform their myriad of complex tasks involved in host homeostasis and consequently health and disease. Adding support to this contention, Jacka et al. (2017) conducted a clinical trial which demonstrated that adherence to a modified Mediterranean diet resulted in significantly greater improvement in depression ratings from baseline compared to a social support control group.

While diet is one way through which a host can modulate their GM, microbes are themselves able to influence eating behaviors of their host (Alcock et al., 2014). Microbes in the gut must cooperate and share limited resources (space and nutrients) to promote stable coexistence and ecological diversity (Allen & Nowak, 2013). This means that these microorganisms are under selective pressure to ensure their own survival and must therefore compete for available resources (Hibbing, Fuqua, Parsek, & Peterson, 2010). As such, microbes are proposed to manipulate the eating behavior of the host by either generating cravings for foods that they thrive on or those which suppress their competitors, or by influencing mood which leads to the intake of foods that enhance that species' fitness (Alcock et al., 2014; Leitao‐Goncalves et al., 2017).

#### 1.2.1 Dietary‐derived short‐chain fatty acids

A continued loss of fiber from the Western diet will inevitably lead to continued depletion of short‐chain fatty acids (SCFAs; Broussard & Devkota, 2016) with downstream effects on the development and perpetuation of psychological illnesses through their immunoregulatory effects (Rogers et al., 2016). SCFAs (acetic, butyric, and propionic acids in particular) are one of the main metabolites of GM (Carabotti et al., 2015; Smith et al., 2013). They are the end products of dietary fiber fermentation and have been shown to have many beneficial effects on host health (Bourassa, Alim, Bultman, & Ratan, 2016; den Besten et al., 2013).

In the brain, SCFAs demonstrate neuroprotective properties (Sun et al., 2015) with butyrate in particular having a protective effect on psychological and neurodegenerative disorders (Bourassa et al., 2016). Peripherally, SCFAs are believed to influence the size and function of regulatory T cells which play a crucial role in regulating inflammation and immune homeostasis (Hakansson & Molin, 2011; Smith et al., 2013). Additionally, SCFAs (together with enzymes also produced by the GM) enhance intestinal barrier functioning through their regulation of tight junction (TJ) proteins (e.g., Anderson et al., 2010; Bischoff et al., 2014; Peng, Li, Green, Holzman, & Lin, 2009). Abnormal intestinal permeability (leaky gut) results in increased translocation of toxins and GM across the epithelial barrier which consequently trigger an inflammatory immune response that can dysregulate ENS and systemic immune functioning (Berkes, Viswanathan, Savkovic, & Hecht, 2003; Carabotti et al., 2015; Fasano, 2012; Smith et al., 2013). This immune response is believed to be the instigator of resultant symptom expression including psychological disorders such as depression (e.g., Maes et al., 2013; Mulak & Bonaz, 2015; Sheedy et al., 2009).

Also via their influence on TJ proteins, SCFAs are believed to regulate the permeability of the blood–brain barrier (BBB; Braniste et al., 2014). Dysregulation of the BBB has been associated with neuropsychological conditions including Alzheimer's disease (Kuhnke et al., 2007) and autism (Fiorentino et al., 2016). Schoknecht and Shalev (2012) suggest that depression and schizophrenia may also be related to BBB dysfunction. Although further research is needed, these associations are highly plausible given the BBB is responsible for regulating access of circulating macromolecules and potential neurotoxins to the brain (Fiorentino et al., 2016; Patel & Frey, 2015). As evidence of microbial involvement, Braniste et al. (2014) found that germ‐free (GF) mice (those devoid of bacterial colonization and therefore lacking conventional gut flora) have increased BBB permeability compared to specific pathogen‐free (SPF) mice that have conventional GM colonization free of any known pathogens. Colonization of GF mice with known SCFA‐producing bacterial strains (*Clostridium tyrobutyricum* and *Bacteroides thetaiotaomicron*) was found to normalize BBB function (Braniste et al., 2014). There is also evidence to suggest that SCFAs are involved in glucose metabolism, reducing adiposity, appetite regulation, and energy homeostasis (Byrne, Chambers, Morrison, & Frost, 2015; Chambers et al., 2015; Kondo, Kishi, Fushimi, Ugajin, & Kaga, 2009; Morrison & Preston, 2016).

### 1.3 Behavior

Studies using GF mice have provided the greatest depth of information regarding the influence of GM on host behavior. Such preclinical work gives useful insights into the physiological mechanisms through which the BGMA functions. For example, GF mice demonstrate altered expression of brain‐derived neurotrophic factor (BDNF) and SCFA while also exhibiting altered HPA axis functioning, anxious and depressive behaviors, and social functioning (Arentsen, Raith, Qian, Forssberg, & Diaz Heijtz, 2015; Luczynski et al., 2016; Neufeld, Kang, Bienenstock, & Foster, 2011; Sudo et al., 2004). In both animal and human models, manipulation of GM has also been demonstrated to alter levels of stress hormones corticotropin‐releasing factor (CRF) and cortisol (Yarandi, Peterson, Treisman, Moran, & Pasricha, 2016). Many of these abnormalities have been shown to be rectified by colonization with the feces from SPF mice or with specific probiotics (e.g., Bercik et al., 2011; Desbonnet et al., 2010; Sudo et al., 2004). However, Bravo et al. (2011) found that ingestion of probiotics was only beneficial in mice which had an intact vagus nerve, demonstrating the importance of the vagal pathway in brain–gut communication. Additionally, Sudo et al. (2004) demonstrated that recolonization was only effective if it occurred within a critical period, providing evidence for a fundamental role of GM in the development of crucial systems involved in behavioral outcomes.

A recently proposed way in which GM may manipulate host behavior is via their relationship with personality traits. Kim et al. (2018) found an increased abundance of *Gammaproteobacteria* in those with high neuroticism, as well as those with low extraversion. Low conscientiousness was associated with an increased abundance of *Proteobacteria* and a decreased abundance of *Lachnospiraceae* while those with high levels of openness demonstrated greater phylogenic diversity and richness (Kim et al., 2018). As this was the first study to have investigated the link between GM and personality directly, further research is required to elucidate these relationships. The relationship between personality and GM presents as an intriguing area of exploration, given that personality traits have a strong association with behavioral patterns in addition to physiological and psychological health outcomes (e.g., Ferguson, 2013; Kim et al., 2018; Srivastava & Das, 2015).

Additional evidence linking GM composition and behavior comes from the study of patients following gastric bypass surgery. Behavioral changes following gastric bypass surgery include patients feeling less hungry and having a preference for healthier foods (Behary & Miras, 2015) which is likely related to changes in neural responses to food (particularly high‐calorie foods) in key areas of the mesolimbic reward pathway (Ochner et al., 2011, 2012). It is tempting to speculate that these changes are associated with the compositional changes in GM following gastric bypass surgery (Furet et al., 2010; Liou et al., 2013; Zhang et al., 2009). Additionally, improvements in quality of life and levels of depression have been shown to persist two years after surgery (Karlsson, Sjostrom, & Sullivan, 1998; Mokhber, Shaghayegh, Talebi, & Tavassoli, 2016). These improvements may be the result of reduced adipose tissue which has downstream effects on GM and their role in inflammation and other related functions. While causational evidence is currently unavailable, correlational research linking GM and mood (Jiang et al., 2015) suggest that changes in GM composition following gastric bypass surgery may also have a direct influence on mood. Improvements in mood may consequently encourage healthier food choices and eating behaviors (Christensen & Brooks, 2006), exemplifying the potential for a cyclical relationship involving diet, GM, and mood that is beneficial to overall health.

Given the possible role of GM in eating behaviors, and their ability to influence hunger and satiety (Cani et al., 2009) and neuropeptide and endocrine regulation (Holzer & Farzi, 2014), a relatively new line of inquiry has emerged investigating GM involvement in eating disorders which are traditionally recognized as psychological disorders (Kleiman et al., 2015; Lam, Maguire, Palacios, & Caterson, 2017). Associations between GM composition and eating disorder psychopathology were also found by Kleiman et al. (2015), further suggesting that the GM play a role in the psychology of food choice and eating behaviors. Given that diet is a key determinant of GM composition and that eating disorders are categorized by extreme dietary changes, the GM present as a logical target for inclusion in multifaceted intervention.

While this paper focuses mainly on unconscious mechanisms underlying the relationship between GM and psychological outcomes (such as interoceptive processes and neurotransmitter production), it is acknowledged that conscious mechanisms also have potential psychological implications. For example, gastrointestinal symptoms can be noticeably unpleasant and, particularly in IBS sufferers, can lead to impairment in daily functioning (Ballou, Bedell, & Keefer, 2015), anxiety and depression (Roohafza et al., 2016), avoidance behaviors (Van Oudenhove et al., 2016), and poor quality of life (Canavan, West, & Card, 2015). Conscious mechanisms can also lead to positive psychological outcomes as exemplified by patients following gastric bypass surgery. For example, noticeable changes in body composition can result in more positive body image which in itself is related to psychological well‐being, particularly after body contouring surgery (Jumbe, Hamlet, & Meyrick, 2017; Sarwer & Steffen, 2015; Song et al., 2016). These changes can then encourage long‐term weight loss maintenance behavior (Palmeira et al., 2010).

### 1.4 Neurotransmitters

**Figure 1:**
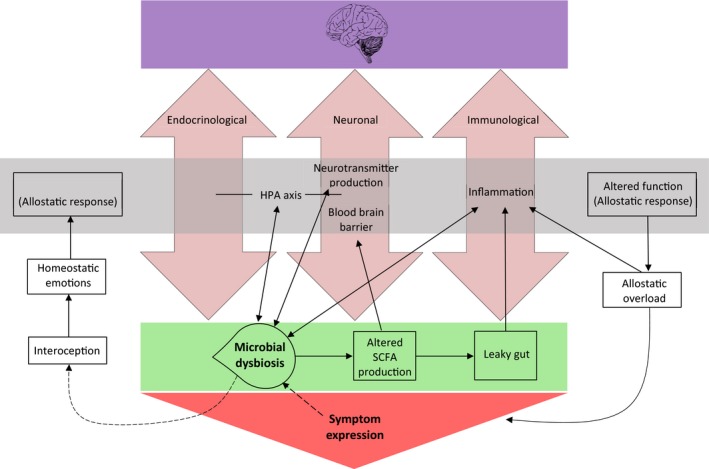
Factors influencing the multidirectional communication between the brain and the gut. Double‐headed arrows demonstrate a bidirectional relationship, with broken arrows demonstrating proposed but not yet established relationships. The figure demonstrates the three main well‐established pathways of communication between the brain and the gut, being endocrinological, neuronal, and immunological. The figure also illustrates the bidirectional relationships between microbial dysbiosis and the HPA axis, neurotransmitter production, the function of the blood–brain barrier, and inflammation which are believed to alter their functioning as an allostatic response to homeostatic emotions. It is proposed that microbial dysbiosis itself is able to be detected via the interoceptive system which then triggers these homeostatic emotions

Perhaps the most obvious association between GM and psychological illnesses is the ability of GM to manipulate the production and action of several key neurotransmitters (e.g., Anderson & Maes, 2015; Lyte, 2011; O'Mahony, Clarke, Borre, Dinan, & Cryan, 2015). GM regulate the metabolism and concentration of amino acids which serve as precursors for several neurotransmitters including gamma‐aminobutyric acid (GABA), serotonin, melatonin, and dopamine, among others (Clarke et al., 2014; Evrensel & Ceylan, 2015; Jenkins, Nguyen, Polglaze, & Bertrand, 2016; Zagajewski et al., 2012). As such, it is highly plausible that GM are able to influence brain chemistry, which consequentially regulates cognition, mood, and behavior. As depicted in Figure 1, demonstrating bidirectionality, GM can also be directly affected by neurochemicals which alter bacterial growth and pathogenicity (Lyte, 2011). Table 1 displays some of the key neurotransmitters synthesized by GM whose dysregulation is associated with psychological disorders. While neurotransmitters produced in the gut may not directly influence brain chemistry as they do not pass through the BBB, they are able to influence the CNS through mechanisms including direct stimulation of the vagus nerve, as well as using indirect circulatory and immune pathways (Sampson & Mazmanian, 2015). For example, tryptophan, the precursor molecule to serotonin (which is itself the precursor to melatonin), is able to pass through the BBB and as such it is likely that metabolites of GM directly influence brain chemistry (Sampson & Mazmanian, 2015).

**Table 1:** Gut bacteria associated with the synthesis of key neurotransmitters

Genus/species	Neurotransmitter (precursor)	CNS effect	Peripheral effect	Psychiatric conditions related to dysregulation	References
*Candida; Streptococcus; Escherichia; Enterococcus; Lactobacillus bulgaricus*	Serotonin (tryptophan)	Motor control, cerebellar regulation, synaptogenesis, addiction, emotion, memory, stress	Circadian rhythm, gut motility, body temperature, visceral pain, appetite, modulation of immune response	Depression, IBS, autism, Down's syndrome	Arreola et al., (2015), Gulesserian, Engidawork, Cairns, and Lubec (2000), Halford and Blundell, (2000), Hood et al. (2006), Jenkins et al. (2016), Leonard, (2010), Mazzoli and Pessione (2016), Marks et al. (2009), Meneses and Liy‐Salmeron (2012), Müller and Homberg (2015), Rogers et al. (2016), Stasi, Rosselli, Zignego, Laffi, and Milani (2014), Warren and Singh, (1996), Whitaker‐Azmitia (2001)
*Corynebacterium glutamicum; Lactobacillus plantarum; Lactobacillus paracasei; Lactobacillus lactis; Brevibacterium lactofermentum; Brevibacterium flavum*	l‐glutamate	Excitatory, brain development, synaptic plasticity		Generalized anxiety disorder, depression, bipolar, schizophrenia, neurodegeneration	Abdou et al. (2006), Boonstra et al. (2015), Cherlyn et al. (2010), Femenía, Gómez‐Galán, Lindskog, and Magara (2012), Hyland and Cryan (2010), Meldrum (2000), Yoto et al. (2012)
*Lactobacillus; Bifidobacterium; Escherichia coli; Pseudomonas*	GABA (l‐glutamate)	Inhibitory, anxiolytic	Myorelaxant, moderates intestinal motility, gastric emptying, gastric acid secretion, and inhibits GI carcinogens and tumor growth		
*Bacillus, Serratia, E. coli*	Dopamine (l‐Dopa)	Reward‐motivated behavior, motor behavior, cognition, emotion	Stimulates exocrine secretion, inhibits gut motility, and modulates sodium absorption and mucosal blood flow	Schizophrenia, Parkinson's disease, depression, anxiety, addiction	Di Chiara and Bassareo (2007), Eisenhofer et al. (1997), Freestone (2013), Grace (2016), Lyte (2011), Meyer and Feldon (2009), Scheperjans et al. (2015), Shishov, Kirovskaya, Kudrin, and Oleskin (2009)
*Bacillus; E. coli; Saccharomyces*	Norepinephrine (dopamine)	Stress hormone, attentiveness, emotion, sleep, learning	Mediates growth and virulence of potentially pathogenic bacteria	Depression, schizophrenia	Freestone (2013), Moret and Briley (2011), Yamamoto and Hornykiewicz (2004)
(dependent on tryptophan production and serotonin synthesis)	Melatonin (serotonin)	Circadian rhythm, mood	Gastrointestinal function, protects against gut permeability, anti‐inflammatory, antioxidant, analgesic	IBS, multiple sclerosis, autism, Alzheimer's, mood disorders	Fornaro, Prestia, Colicchio, and Perugi (2010), Ghorbani, Salari, Shaygannejad, and Norouzi (2013), Ortiz, Benítez‐King, Rosales‐Corral, Pacheco‐Moisés, and Velázquez‐Brizuela (2008), Veatch, Goldman, Adkins, and Malow (2015), Wong, Yang, Song, Wong, and Ho (2015)

### 1.5 Interoception and allostatic responses

It is currently unknown whether the interoceptive system is able to detect microbial dysbiosis (imbalances resulting from the under‐ or overabundance of certain microbial species; DeGruttola, Low, Mizoguchi, & Mizoguchi, 2016), but considering that gut microbes are an essential part of human physiology which moderate several homeostatic emotions (Craig, 2002; Mayer, Naliboff, & Craig, 2006; Noakes, 2012; Paulus & Stein, 2010) it is a strong possibility. Homeostatic emotions are background emotions that may or may not enter conscious awareness but influence an individual's energy levels, mood, and disposition (Mayer et al., 2006). Signals from internal organs, particularly the gut, continuously communicate with various regions of the brain including the limbic system, autonomic and neuroendocrine centers in the hypothalamus, brainstem, and cortex (Craig, 2002; Holzer & Farzi, 2014; Mayer & Tillisch, 2011). It is plausible that through the process of interoception, GM are able to influence human cognition, emotion, and mood through their involvement in systemic functioning through their various metabolites (Holzer, 2017; Paulus & Stein, 2010). As such, increasing rates of disease might be explained by the allostatic load hypothesis (McEwen, 1998). The allostatic load hypothesis proposes that rather than having a stable set point, body systems have a range of set points allowing them to actively adapt to environmental and internal states. Allostatic load is a term used to refer to the cumulative cost of allostasis to the body (“wear and tear”; McEwen & Wingfield, 2003). While adaptive in the short term, allostasis can become maladaptive, leading to disease, when allostatic measures are required to vary widely and frequently, or are at extreme values for long periods of time (James, 2013). Additionally, allostatic systems can become dysfunctional when they lose their ability to change or regulate change (James, 2013). The result of either of these scenarios is allostatic overload which can lead to symptom expression, as depicted in Figure 1 (Berger, Juster, & Sarnyai, 2015; McEwen, 2005; McEwen & Wingfield, 2003). The fact that research has failed to define the precise composition of a healthy GM due to immense interindividual differences (Lloyd‐Price, Abu‐Ali, & Huttenhower, 2016) suggests that the GM may in fact be the most allostatic system within the body. Microbial dysbiosis then could be considered an extreme and prolonged shift away from what would be considered a relatively healthy composition which loses its ability to regulate change in various other host systems and functions. It is perhaps this dysregulation that manifests itself in psychological illness.

Figure 1 depicts the three overarching pathways (endocrinological, neuronal, and immunological) of bidirectional communication between the brain and the gut, each of which is altered during a state of microbial dysbiosis. Although it remains unconfirmed, it is proposed that microbial dysbiosis can be detected via interoception which ultimately leads to altered functioning of factors (e.g., inflammation) that mediate these pathways. These alterations are believed to be an allostatic response to a deviation from a “typical” microbiome, which over time, leads to symptom expression as a result of allostatic overload. The bidirectional relationship between microbial dysbiosis and the factors which alter the three main communication pathways between the gut and the brain illustrate the multidirectional nature of the BGMA.

## 2 GM THROUGH THE LENS OF A CASE FORMULATION FRAMEWORK

It is not the intention of this paper to propose that the BGMA must be at the forefront of consideration for each and every client. Instead, it is proposed that the relevance of the BGMA is determined on a case‐by‐case basis. The role of the BGMA in a client's presenting problem may be less relevant when there are clear social and emotional etiological factors, such as the presence of significant stressors for a client presenting with anxiety or depression. However, it is worth noting that stress can alter the composition of a person's GM (Bailey et al., 2011), which may or may not be clinically relevant, but should be considered if comorbidities are present. It may also be less relevant for clients who respond well to traditional psychological treatments such as cognitive behavioral therapy. Alternatively, cases in which the role or the BGMA may be more pertinent are when social and emotional etiological factors are less clear or absent, and also for clients who do not respond well to conventional psychological approaches. In cases where a treating clinician considers investigation of the GM to be appropriate, clients should be referred for stool sampling and analysis and an open dialogue established between the clinician and the microbiologist performing the analysis. The following information serves to highlight possible associations between GM and each of the Four Ps.

### 2.1 Predisposing

As part of their first line of questioning, mental health professionals attempt to explore a client's family history of psychological illness to establish whether that individual has an underlying genetic predisposition (vulnerability) to developing a psychological condition(s). Genetic predisposition to a multitude of psychological conditions has been well established (Hyman, 2000). While research into the role of GM in genetic predisposition is scarce, several lines of evidence suggest that GM may play an integral part in a person's vulnerability to the development of psychological illnesses. Firstly, host genetics have been demonstrated to influence the composition and metabolic activities of GM (Goodrich et al., 2014; Ussar, Fujisaka, & Kahn, 2016) which have important consequences on host physiology, brain development, and health (e.g., Krishnan, Alden, & Lee, 2015; Sekirov, Russell, Antunes, & Finlay, 2010). However, the specific mechanisms behind this relationship remain unclear (Dąbrowska & Witkiewicz, 2016).

#### 2.1.1 Vertical transmission

Additionally, there is evidence to suggest that much like genetics are passed down from parents to offspring, GM are vertically transmitted from mother to infant (e.g., Asnicar et al., 2017; Mueller, Bakacs, Combellick, Grigoryan, & Dominguez‐Bello, 2015). The transmission of microbiota from mother to infant during birth represents the most important point of microbial colonization in the infant gut, which continues over the first three years of life (Yatsunenko et al., 2012). This critical establishment period of GM is in line with the critical development period of the human host (Rea et al., 2016). It is during this time that GM play key roles in the development of the CNS, HPA axis, and immune system (Borre et al., 2014; Cox et al., 2014; Furusawa et al., 2013; Houghteling & Walker, 2015). As such, aberrations in typical colonization of GM during this critical period may also result in abnormal development of these key systems (Tamburini, Shen, Wu, & Clemente, 2016). Factors resulting in aberrations to conventional microbial colonization of the gut during this period, such as birth by cesarean section and antibiotic treatment during infancy, have been associated with increased rates of chronic and atopic diseases (Kolokotroni et al., 2012; Sevelsted, Stokholm, Bonnelykke, & Bisgaard, 2015; Vangay, Ward, Gerber, & Knights, 2015). A recent study by Polidano, Zhu, and Bornstein (2017) highlights the microbiota as a potentially important factor in the negative relationship they found between cesarean birth and a range of cognitive outcomes compared to those born vaginally.

There is a growing consensus that maternal GM may have long‐term health consequences for the child (Stanislawski et al., 2017). It is therefore reasonable to suggest that vertically transmitted GM may act as a mechanism for intergenerational predisposition to psychological disorders. Further research is required as it is difficult to determine whether these intergenerational patterns are due to the vertical transmission of GM or whether they are due to learned behaviors and lifestyle factors shared among family members. This is evidenced by same‐household members showing a higher similarity of GM composition to those outside of the household (Abeles et al., 2016; Song et al., 2013; Yatsunenko et al., 2012).

#### 2.1.2 Aging

Aging, in and of itself, can also predispose an individual to several psychological illnesses. For example, neurodegenerative illnesses, such as Alzheimer's disease, are considered an evolutionary accident occurring as a result of increased longevity (Giunta et al., 2008; Gluckman et al., 2011; Niccoli & Partridge, 2012). There is evidence to suggest that psychological disorders are also more prevalent in the elderly (e.g., Andreas et al., 2017). A contributing factor toward the increased prevalence of disease in the elderly is that the normal aging process is associated with compositional changes and reduced diversity of GM (Biagi et al., 2010). In parallel, normal aging is characterized by chronic low‐grade inflammation, a phenomenon commonly referred to as “inflammaging” (Franceschi et al., 2007). It is believed that this inflammation is, at least in part, attributable to alterations in GM (Buford, 2017). Additionally, aging is associated with changes in the serotonergic system which is also believed to contribute to increased prevalence of psychological disorders in the elderly (O'Mahony et al., 2015). The serotonergic system is regulated by GM (Table 1), therefore making it vulnerable to compositional and metabolic changes (O'Mahony et al., 2015; Rogers et al., 2016).

Given the sheer complexity of the systemic functioning of the human body, there are likely to be several other processes through which GM may be involved in predisposing individuals to the development of psychological illnesses. Further research into how GM influence predisposition to psychological illness will be useful in informing preventative strategies to circumvent the growing burden of such conditions. However, GM not only play a role in predisposing an individual to certain psychological illnesses as highlighted above, but also in the onset and maintenance of those negative health outcomes.

### 2.2 Precipitating and perpetuating

While psychologists tend to focus heavily on social and environmental factors involved in the onset and maintenance of psychological disorders, biological factors, such as GM composition, also contribute to these stages of disease. The maintenance of a diverse microbial ecosystem in the gut is essential for optimal host function (Moloney et al., 2014; Sekirov et al., 2010). On the other hand, reduced diversity and microbial dysbiosis have been implicated in various psychological, neurological, metabolic, functional gastrointestinal disorders, and autoimmune disease states (Blumstein, Levy, Mayer, & Harte, 2014). These include, but are not limited to, IBS (Jeffery et al., 2012; Tana et al., 2010), autism (Finegold et al., 2002), schizophrenia (Castro‐Nallar et al., 2015), myalgic encephalomyelitis/chronic fatigue syndrome (ME/CFS; Fremont, Coomans, Massart, & Meirleir, 2013; Wallis, Butt, Ball, Lewis, & Bruck, 2016), multiple sclerosis (Jangi et al., 2016), dementia (Alkasir, Li, Li, Jin, & Zhu, 2017), stress (Knowles, Nelson, & Palombo, 2008), anxiety (Burch, 2016), depression (e.g., Jiang et al., 2015), obesity (Ley, Turnbaugh, Klein, & Gordon, 2006), diabetes (Larsen et al., 2010), coronary artery disease (Cui, Zhao, Hu, Zhang, & Hua, 2017; Emoto et al., 2017), and cancer (particularly colorectal cancer; Gagniere et al., 2016; Garrett, 2015). Current findings linking GM and disorders are, at this stage, associative and causative links are yet to be established. Additionally, it is unknown whether alterations in GM composition are the cause or consequence of disease. Given available evidence however, it is likely that this is a bidirectional, and cyclical, relationship as depicted in Figure 1.

#### 2.2.1 Stress

Stress has long been recognized as both a precipitating and perpetuating factor to various psychological conditions (Anisman & Zacharko, 1982; Corcoran et al., 2003). Given the crucial role of GM in the development and functioning of the HPA axis (Sudo, 2012), as well as their modulation of stress hormones CRF and cortisol (Carabotti et al., 2015), the involvement of GM in the stress response is increasingly evident. This is further demonstrated by the ability to transfer stress‐prone phenotypes from one mouse to another via fecal transplantation (Collins, Kassam, & Bercik, 2013). Stress also activates an inflammatory response via the promotion of inflammatory cytokines (Liu, Wang, & Jiang, 2017). Given that inflammation is recognized as underlying many psychological and neurodegenerative disorders (Almond, 2013; Miller & Raison, 2016; Rea et al., 2016), stress then appears to play a role in both the etiology and maintenance of psychological illness via biological pathways, all of which are regulated by the GM. While this is reflective of a bottom‐up process whereby GM influence stress, substantial evidence also suggests the occurrence of a top‐down process through which stress regulates GM composition (Bailey et al., 2011; Gur, Worly, & Bailey, 2015; Knowles et al., 2008). The fact that both top‐down and bottom‐up processes have been well established demonstrates the complex cyclical and multidirectional relationship between GM, stress, and psychopathology.

#### 2.2.2 Socioeconomic status

There is an established association between low SES and several factors negatively affecting health, one of which is poor diet (Darmon & Drewnowski, 2008; Shahar, Shai, Vardi, Shahar, & Fraser, 2005). Given that diet is the strongest environmental contributor to a properly functioning GM (e.g., De Filippo et al., 2017; Garcia‐Mantrana et al., 2018), it is possible that the composition and/or metabolic activities of the GM is one of the mediating factors of the relationship between low SES and mental health outcomes. Another factor associated with low SES is lower educational achievement (Sirin, 2005) which may also be mediated by diet‐related changes in GM. Individuals who eat a poor‐quality diet (Western diet) have poorer performance on cognitive tasks (Khan et al., 2015) and poorer mental health (Jacka, Kremer, et al., 2011; Jacka, Mykletun, Berk, Bjelland, & Tell, 2011; Markus et al., 1998) compared to those who eat high‐quality diets. In addition, an association has been found between the consumption of a Western diet and decreased left hippocampal volume (Jacka, Cherbuin, Anstey, Sachdev, & Butterworth, 2015) with hippocampal volume being related to cognition (Choi et al., 2016) and mood (Frodl et al., 2006). It is likely the GM mediate this relationship through their production of SCFAs and BDNF which have been found to be involved in neurogenesis and neuronal protection in mouse models (Canani, Di Costanzo, & Leone, 2012; Lee, Duan, & Mattson, 2002; Sun et al., 2015). Ultimately, poorer performance on cognitive tasks and poorer mental health limit a person's educational attainment (Eisenberg, Golberstein, & Hunt Justin, 2009; Fletcher, 2010; McLeod & Fettes, 2007).

### 2.3 Protective

Considering the protective abilities of GM or indeed, specific microorganisms, has the potential to revolutionize the treatment of psychological conditions (Kali, 2016; Mazzoli & Pessione, 2016; Sampson & Mazmanian, 2015). The addition of GM modulation to an individual's treatment plan may be the missing link in explaining and counteracting the alarming increase in the disease burden of common mental disorders.

#### 2.3.1 Lifestyle factors

Healthy eating and exercise have long been promoted as being protective factors against both physiological and psychological conditions. Evidence suggests that one of the physiological mechanisms through which healthy eating and exercise affect health is the influence these factors have on the composition and metabolic activity of GM (Mika et al., 2015; Welly et al., 2016). Essentially, a high‐quality diet and exercise provide the GM with the resources they require to maintain optimal host function. While the influence of diet on GM composition is widely researched, that of exercise on GM receives less attention. However, exercise has been shown to enrich microbial diversity, improve the *Bacteroidetes*‐to‐*Firmicutes* ratio, and support the growth of SCFA‐producing bacteria which have immunomodulatory effects (Monda et al., 2017). This suggests that like diet, the beneficial outcomes of exercise may be mediated by GM which have downstream effects on mental health.

#### 2.3.2 Pre‐ and probiotics

Both pre‐ and probiotics have also been demonstrated to have psychotropic like effects in healthy volunteers as well as those suffering from conditions such as depression and chronic fatigue syndrome (CFS; Akkasheh et al., 2016; Messaoudi et al., 2011; Rao et al., 2009). Probiotics showing positive effects on mental health are referred to as psychobiotics (Dinan, Stanton, & Cryan, 2013). Studies have demonstrated that various probiotic formulations (mostly including *Lactobacillus* and *Bifidobacterium* species) have the ability to improve mood in healthy (no reported diagnoses of allergic, neurological, or psychological conditions) men and women (Benton, Williams, & Brown, 2007; Messaoudi et al., 2011; Steenbergen, Sellaro, Hemert, Bosch, & Colzato, 2015). In a placebo‐controlled study, Yamamura et al. (2009) found a probiotic formulation to improve sleep efficacy and number of awakenings (as measured by actigraphy) in an elderly (60‐ to 81‐year‐old) sample. Moreover, a study using fMRI revealed altered activity in brain regions responsible for emotion and sensation processing in women following four weeks of probiotic formulation intake compared to women who received a placebo (Tillisch et al., 2013). Also in a placebo‐controlled study, participants who took a prebiotic (Bimuno‐galactooligosaccharides) daily for three weeks showed significantly lower salivary cortisol levels and decreased attentional vigilance to negative versus positive information (Schmidt et al., 2015). These findings were similar to those of a study that involved the administration of an SSRI (Murphy, Yiend, Lester, Cowen, & Harmer, 2009).

#### 2.3.3 Fecal microbial transplant

The increasing popularity of fecal microbial transplant (FMT) in treating various conditions including but not limited to GI disorders (Brandt & Aroniadis, 2013), Parkinson's disease (Ananthaswamy, 2011), autism (Aroniadis & Brandt, 2013), and ME/CFS (Borody, Nowak, & Finlayson, 2012) is perhaps due to the proliferation of research associating microbial dysbiosis to a range of disorders. In humans, the efficacy of FMT has been shown for conditions such as ulcerative colitis (Shi et al., 2016), but has not yet been demonstrated in treating psychological conditions. It does however offer a promising avenue given strong evidence suggesting a role of GM in the pathogenesis of psychological conditions (Evrensel & Ceylan, 2016). Animal models suggest that FMT is an effective way to ameliorate abnormal physiology and function (e.g., Sudo et al., 2004); however, clinical trials are required to demonstrate whether this approach is equally effective in humans. Additionally, further research is required into the possible risks associated with FMT. While FMT has promising therapeutic potential, Alang and Kelly (2015) present a case study of a patient who developed obesity following FMT treatment from an overweight, but otherwise healthy donor. As GM are associated with numerous physiological and psychological conditions, FMT could theoretically result in the transference of any such condition from donor to recipient (Bunnik, Aarts, & Chen, 2017). As such, it is clear that great care must be taken when screening and selecting potential donors. There is still much to learn about the associations between GM and both physiological and psychological conditions, and therefore, potential long‐term risks of FMT may yet to emerge.

## 3 CRITICISMS OF CONVENTIONAL TREATMENT WITH RESPECT TO GM

Conventional treatment of psychological disorders typically involves pharmacological intervention such as psychotropics and/or other medications to alter brain chemistry (e.g., Bystritsky, Khalsa, Cameron, & Schiffman, 2013; Lieberman et al., 2005). Although beneficial, such treatments may induce undesirable side effects including, but not limited to, nausea, sleep disturbance, weight gain, and sexual dysfunction (Ferguson, 2001) all of which may themselves be a result of microbe‐mediated drug metabolism (Enright, Joyce, Gahan, & Griffin, 2017). Despite the ever increasing reliance on pharmacotherapy (Kallivayalil, 2008; Vozeh, 2003), disease states remain relatively stable which suggests the need for auxiliary treatment options and/or targets which take into account several body systems, including the GM. Many psychotropic drugs, known for their influence on CNS receptor function, also demonstrate antimicrobial effects (Kalayci, Demirci, & Sahin, 2014) which may have unintended consequences on the BGMA. This is particularly true of many SSRIs commonly used to treat depression and anxiety disorders.

In addition to having direct antimicrobial effects, Ayaz et al. (2015) found that sertraline (an SSRI) augments the effectiveness of several antibiotics by increasing their inhibitory zone. As such, chronic use of these drugs can induce potentially deleterious alterations in GM (Macedo et al., 2017). This may partially explain treatment resistance, although further research is needed to support this notion. Likewise, psychological interventions (e.g., cognitive behavior therapy) also target the brain via a focus on cognitions to affect behavioral change. This top‐down process has demonstrated efficacy in the treatment of functional gastrointestinal disorders such as IBS (Boersma et al., 2016; Palsson & Whitehead, 2013); however, research is needed to determine whether purely psychological interventions can enact changes in GM. By continuing to treat various disorders and symptoms through pharmacological intervention without considering the etiology of initial chemical imbalances, it is unlikely that rates of morbidity will decline. The net effect of ignoring etiology at the expense of treatment is therefore an increased burden upon individuals who are affected by disease, and wider society.

## 4 REDEFINING WHO WE ARE

There is currently a shift away from thinking of host–microbe interactions in such binary terms toward appreciating the complexity of the relationship between the two. Binary distinctions between host and microbiota remain useful only in so far as to aid our understanding of the role of GM in psychological well‐being, which is still in its infancy. However, emerging nomenclature such as “holobiont” acknowledges that GM are not a separate entity but are instead an integral and inseparable part of human biology (Schnorr, Sankaranarayanan, Lewis, & Warinner, 2016; Theis et al., 2016). This concept is supported by the coevolution of humans and their microbes. The concept that the ratio of bacterial cells to human cells is approximately 1:1 (recently revised down from previous estimations of 10:1; Sender, Fuchs, & Milo, 2016) pays homage to the importance of respecting GM in the conceptualization of human health and well‐being. This reconceptualization of what makes us human also provides a biological and tangible basis for explaining and treating psychological illnesses that can often be considered abstract.

## 5 CHALLENGES AND THE WAY FORWARD

Understanding the multidirectional relationship between GM and the nervous system is hindered by its inherent complexity (Mazzoli & Pessione, 2016). However, while still in its infancy, interdisciplinary research has uncovered novel ways of conceptualizing disease. While theoretically relevant, research into the BGMA also has important practical implications, offering a more holistic approach to treatment and prevention of psychological illness. While this new field of research is promising, it remains unclear which factors, and in which combination, alter the balance between symptomatic and asymptomatic outcomes.

The sheer number of confounding variables makes it difficult to establish causational links between GM and symptomatology. However, as understanding of the GM advances, so too will research methodology and technology. It is only with continued research into the link between GM and psychological illness that we will be able to elucidate the true extent to which our resident microbes contribute to mental health. As the majority of studies regarding the role of GM in both physiological and psychological health and disease have been conducted using animal models, clinical trials with human samples are imperative to the advancement of knowledge and ultimately practical application.

As the burgeoning research into the relationship between GM and psychological health outcomes gathers momentum, so too does the call to action for psychologists to embrace the microbiome as a potential factor in explaining, treating, and preventing mental illness. This is an important paradigm shift which must occur within the discipline of psychology in order to keep up to date with the most current and complete knowledge of the human body and mind which translates into providing the best possible care for clients. While it is unnecessary and impractical to expect psychologists to develop a detailed understanding of the influence of GM on mental health, it is important that the role of the GM is acknowledged, especially in the absence of clear social and emotional factors. Facilitating this paradigm shift may require change at a “grass roots” level, where psychobiology is better integrated into higher education psychology degrees. Additionally, professional development courses regarding the role of the GM in mental health should be established and promoted to current practicing psychologists who can use this information to provide more complete care for their clients.

The fact that GM are able to influence psychological functioning is an exciting and encouraging prospect, which begs for multidisciplinary approaches to both research and practice. In terms of practical implications, increasing our understanding of the mechanisms that mediate communication processes between GM and host has the potential to inform strategies to limit the damaging aspects of this communication. This will provide new avenues of treatment for a wide range of symptoms and disorders (Freestone, 2013) as well as to promote good health. This will require a substantial shift away from the reductionist approaches that see us working exclusively in our specific field. This is not to suggest that psychologists should become expert in areas outside of their field, but instead to understand and acknowledge that the best way forward is a multidisciplinary approach to the treatment and prevention of mental illness. A shift toward a multidisciplinary, and therefore more holistic, approach will provide an opportunity to better understand the etiology of disease which requires the expertise of several disciplines and a consideration of key body systems, including the GM, as intertwined and inseparable. In light of the evidence research has provided thus far, psychologists working as part of multidisciplinary teams with other professionals such as nutritionists, gastroenterologists, and microbiologists must seriously consider the inclusion of dietary plans, pre‐ and probiotics, and potentially even FMT in the treatment plans of their clients, in conjunction with conventional psychological treatments.

It is not the contention of this paper to claim that ameliorating gut health is the panacea to all psychological disorders and symptomatology. Instead, it is to demonstrate the complex interconnectivity between multiple body systems in disease processes, from etiology through to treatment, and ideally prevention. In support of the call to action by Allen et al. (2017), the discipline of psychology must shift away from its CNS‐centric conceptualization of disease and symptom‐centered disease treatment models toward a multidisciplinary approach to treatment and prevention. This paradigm shift will empower psychologists to better treat and care for their clients. A multidisciplinary approach where healthcare professionals across a variety of disciplines have a united approach to treatment and prevention will also empower the public to better understand and take control of their physiological and psychological health. It is only through this shared awareness that the healthcare community can make inroads into improving the mental health of current and future generations.

## CONFLICT OF INTEREST

None declared.

## Data Availability

Data sharing is not applicable to this article as no new data were created or analyzed in this study.
